# Comparing databases: determinants of sexually transmitted infections, HIV diagnoses, and lack of HIV testing among men who have sex with men

**DOI:** 10.1186/s12889-015-2445-3

**Published:** 2015-11-12

**Authors:** Chantal den Daas, Maaike Goenee, Bouko H. W. Bakker, Hanneke de Graaf, Eline L. M. Op de Coul

**Affiliations:** Centre for Infectious Disease Control, National Institute for Public Health and the Environment, P.O. Box 1, 3720 BA, Bilthoven, The Netherlands; Rutgers, Utrecht, The Netherlands

**Keywords:** Men who have sex with men (MSM), STI, HIV, HIV testing, Sampling methods

## Abstract

**Background:**

Early detection and treatment of STI/HIV are public health priorities. Our objective was to compare characteristics of men who have sex with men (MSM) in Dutch data available in 2010 from EMIS, an international internet survey, Schorer Monitor, a Dutch internet survey, and data from STI- clinic visits, since these might be subject to different and unknown biases.

**Methods:**

Data from Dutch MSM Internet Surveys (EMIS_NL_*N* = 3,787; Schorer Monitor, SMON *N* = 3,602), and 3,800 STI clinic visits (SOAP) were combined into one dataset. We included factors that were measured in all three databases. The socio-demographics included were age (at the time of the survey), zip code, and ethnicity. Behavioural variables included were the number of sexual partners, condom use with last sexual partner, drug use, being diagnosed with STI, being diagnosed with HIV, and HIV testing. Outcomes we investigated were being diagnosed with STI, HIV, and never been tested for HIV.

**Results:**

Logistic regressions showed that determinants for being diagnosed with STI were having more sexual partners, drug use, and having had an HIV test (aORs 1.3 to 17.1) in EMIS and SMON. Determinants for being diagnosed with HIV in all three databases were older age, living in Amsterdam, and having more partners (aORs 1.8 to 4.4). In EMIS and SMON, drug use, non-condom use, and having STI were additional determinants (aORs 1.6 to 8.9). Finally, determinants associated with never been tested for HIV were being younger (only SOAP), living outside of Amsterdam, having fewer partners, no drug use, and no STI (aORs 0.2 to 0.8).

**Conclusions:**

Risk factors from internet surveys were largely similar, but differed from STI clinics, possibly because it involves self-reports rather than diagnoses or because of differences in timing. The difference between the internet surveys and STI clinic data is much less pronounced for having never been tested, suggesting both are appropriate for this outcome. These findings shed light on conclusions drawn from different data sources, as well as the comparability of recruitment strategies, the robustness of risk factors, consequences of phrasing questions differently, and on (policy) implications based on different data sources.

## Background

In 2013, 88 % of the new HIV infections in STI clinics in the Netherlands were diagnosed among men who have sex with men (MSM) [1]. Furthermore, MSM were more at risk for sexually transmitted infections (STI), such as gonorrhoea and syphilis compared to heterosexual men or women [1]. Therefore, MSM are often targeted for prevention activities and research investigating sexual behaviour.

Comparing findings regarding sexual behaviour among MSM obtained from different sources is challenging for a variety of reasons. First, while most surveillance systems provide good data on diagnoses and positivity rates of STI/HIV among MSM, and these systems increasingly include measures of sexual behaviour, these measures are often less readily available and standardized. Second, data on sexual behaviour among MSM are typically based on convenience samples, such as venue-based surveys. Therefore, research might have resulted in mixed influence of socio-demographic characteristics and behavioural risk factors [2–4]. Third, data sources might consist of identical (double participation) or similar (in terms of behaviour) MSM, possibly because of biases in self-selection. Since dating and cruising sites are often used in the recruitment of MSM, highly sexually active MSM are probably overrepresented [2].

In addition, when sexual behaviour is assessed, differences in question phrasing occur, which can result in pronounced shifts in meaning and ultimately answers. For example, in case of reference periods, if a researcher assesses a longer period this could influence participants’ memory of and interpretation of the behaviour. Specifically, assessing larger time frames could lead to the interpretation that researchers are interested in more severe situations [5]. In terms of sexual risk behaviour, assessing longer periods could similarly result in shifts in severity of the reported risk behaviour. Besides language comprehension, other factors can also influence questionnaire answers. Specifically, psychological research has shown that minor changes in response formats, question context, question framing, and questionnaire source can have pronounced influences on answers [6–9].

It has been shown that Dutch participants in the European MSM Internet Survey (EMIS) are somewhat biased towards older, gay identified, HIV positive, and (sexually) active MSM, compared to participating MSM from other countries [10]. Comparing data of several Dutch data sources is helpful to gain insights into which MSM participated. Another reason to compare databases is that different studies found divergent risk factors. An epidemiological review, for instance, showed that having concurrent STI did not influence HIV transmission [11], whereas this effect was observed in clinical studies [12]. Notably, the exclusion criteria of this review specified results obtained from surveys.

Faced with the diversity approaches measuring socio-demographic and behavioural data, an unresolved question is what the effect of using different recruitment methods has on findings, and which findings are stable (comparable and reliable) across studies. The current study compares three databases to gain insights into differences between study populations, measurement methods, and the robustness of risk factors associated with being diagnosed with an STI or HIV, or never been tested for HIV. Lack of HIV testing is increasingly becoming an important topic of investigation, as up to 90 % of new HIV infections could be transmitted by people unaware of their infection [13]. Moreover, some MSM who were never tested for HIV showed risk behaviour and were at risk of contracting HIV. With insights on the (lack of) differences, the findings of studies using these kinds of databases can be interpreted with more certainty and recommendations for future studies and targeted control policies can be made.

## Methods

### Databases

This study was a secondary analysis of three anonymized databases described below. The European MSM Internet Survey (EMIS) is a multilingual, cross-sectional, online evaluation of HIV prevention needs of MSM in 38 countries. In total 3,787 men living in the Netherlands completed the survey from June 4^th^–August 31^st^ 2010. MSM were recruited predominantly via instant messages on PlanetRomeo, Gaydar, and e-mails to Schorer Monitor participants, as well as via banners on websites that are frequently visited by MSM, through gay community organizations, and by using printed materials. An extensive description of the survey methods can be found elsewhere [14, 15]. EMIS was approved by the Research Ethics Committee of the University of Portsmouth, United Kingdom (REC application number 08/09:21). Participants had to confirm that they had read the introductory text and consented to participate before proceeding to the questions.

The Schorer Monitor (SMON) is a yearly Dutch survey (up to 2011), investigating health, well-being, and sexuality among MSM in the Netherlands. In 2010, the SMON was filled out by 3,602 MSM; from March 22^th^–May 2^nd^. Recruitment was done via banners, printed materials, snowballing (men could invite three friends to participate), and as the SMON was a yearly initiative; men that participated in 2009 were invited to participate again [16]. Participants read an introductory text, containing information about the goals of the survey and privacy information. After this information a button was presented with ‘I will participate’, which routed them to the questions.

SOAP (Dutch abbreviation for ‘SOA Peilstation’ meaning STI registration system) is a database, containing information on STI consultations, -tests and -diagnoses from STI clinics in the Netherlands for surveillance purposes, but is more limited regarding behavioural information [1]. In 2010, 19,579 MSM STI consultations took place. We selected 3,800 sequential cases from an uninterrupted period, starting January 4^th^ and ending March 17^th^ (the 3,800^th^ case), to attenuate the chance of double cases (MSM visiting the STI clinic more than once in 2010) in our analyses. Ethical approval for the study was not necessary following Dutch law as the study used anonymous patient data collected for routine surveillance [17].

#### Measures

We included factors that were measured in all three databases in our comparison; as such, SOAP was the limiting database. Socio-demographics included age (at the time of the survey), zip code, and ethnicity. Behavioural variables included the number of sexual partners, condom use with last sexual partner, drug use, being diagnosed with STI, being diagnosed with HIV, and HIV testing. Differences between questions in the databases were present (Table 1). For example, in both EMIS and SMON data on STI/HIV is self-reported. In SOAP, laboratory diagnoses were available for STI/HIV, as well as self-reported HIV infection.

**Table 1:** Questions

Variable	EMIS	SMON (translation)	SOAP (translation)
Age	How old are you?	What is your age?	Year of birth
Residence	What are the first two digits of your home post-code?	What are the first three digits of your home post-code?	Post-code (4 digits)
Ethnicity	Which country were you born in?	Which cultural background did you grow-up with?	Ethnic group
Number of partners	How many different non-steady male partners have you had sex with in the last 12 months?	How many different non-steady male partners have you had sex with in the last 6 months?	How many persons have you had sexual contact with in the last 6 months?
Condom use last partner	Did he [the non-steady sex partner you most recently had sex with] use a condom during AI?	Did he [the non-steady sex partner you most recently had sex with] use a condom during AI?	Did you use condom(s) during the last sexual contact?
Drug use	When was the last time you consumed X? (recode: yes/no w/i 6 mo)	X – How often did you use in the last 6 months?	Intravenous drug use
STI	When were you last diagnosed with X? (recode: y/n w/i 12 mo)	In the last 12 months, did you receive a positive STI diagnosis? (X yes/no)	Laboratory diagnosis
HIV	Have you ever received an HIV test result? (status in answer options)	Have you ever been tested for HIV? + What was the result?	Diagnosis/Earlier HIV-test

*AI* anal intercourse, *STI* sexually transmitted infection, *HIV* human immunodeficiency virus

Another notable difference between databases is the assessment of number of sexual partners (Table 1 contains the analysed variables). In SOAP this variable includes female and steady partners, besides casual male partners. Reducing the difference between SOAP, EMIS, and SMON could be accomplished by including steady partners to the non-steady male partner measures. We have not done this as EMIS and SMON measured steady male partners differently. Specifically, EMIS considered the number of steady male partners that MSM had sex with over the last 12 months (e.g., more than 10 was an answer category), whereas SMON assessed sex with a steady male partner over the last 6 months (answer categories yes-no). Therefore, we decided to keep EMIS and SMON as similar as possible, thus not including steady and female partners.

#### Analyses

Uni- and multivariable logistic regression analyses were conducted to investigate associations between the outcomes, socio-demographic and behavioural factors for each database. The outcomes were being diagnosed with (one or more) STI, being diagnosed with HIV, and never been tested for HIV. Additionally, we analysed the interactions between the variables and databases to assess whether the effect of the variables on the outcomes differed significantly between databases.

We recoded residence to Amsterdam and a rest category, as numbers in the other cities were too limited to analyse separately and showed similar patterns. We also calculated a compound variable for drug use and being diagnosed with STI within the last 6/12 months. Recoded answer options for the two questions were ‘No’ (I did not have an STI/I did not use any drugs), ‘Yes one’ (I was diagnosed with one STI/I used one kind of drug), and ‘Yes more than one’ (I was diagnosed with more than one STI/I used more than one kind of drugs). Backward selection was performed, a priori including all variables for the likelihood ratio test. All statistical analyses were performed using IBM SPSS for Windows 19, and a *p*-value of < 0.05 was considered statistically significant.

## Results

### Characteristics of study populations

Baseline characteristics of the databases are summarized in Table 2. Due to the large number of participants, even small differences became significant and all variables differed between databases. Notably, EMIS had more non-Dutch participants (22.4 % vs 12.7 % and 17.9 % in SMON and SOAP respectively), which can be explained by the European nature of this survey. This survey was available in 24 languages, and promoted internationally; therefore, this survey obtained a more ethnic diverse sample. This is also clear from the distribution of ethnicities, as fewer participants are from the four biggest minority groups in the Netherlands (e.g., Turkey, Morocco, Surinam, and the Netherlands Antilles; 5.8 % of the non-Dutch participants in EMIS vs 19.9 and 20.9 %, in SMON and SOAP).

**Table 2:** Baseline characteristics of the study population, *N* (%) unless indicated otherwise, stratified by database

		EMIS	SMON	SOAP
Age	Range	14–87	14–86	16–76
	Mean (SD)	39.81 (12.34)	38.36 (13.51)	37.99 (11.66)
	Median	40	39	38
Residence	Amsterdam	1019 (26.9)	706 (19.6)	1433 (37.7)
	Rotterdam	185 (4.9)	175 (4.9)	215 (5.7)
	The Hague	167 (4.4)	138 (3.8)	170 (4.5)
	Utrecht	122 (3.2)	144 (4.0)	93 (2.4)
	Other areas	1881 (49.7)	2280 (63.3)	1785 (47.0)
	Missing	413 (10.9)	159 (4.4)	104 (2.7)
Ethnicity	Dutch	2833 (74.8)	3131 (86.9)	3114 (81.9)
	Not Dutch	849 (22.4)	458 (12.7)	679 (17.9)
	Missing	105 (2.8)	13 (0.4)	7 (0.2)
Not Dutch	Turkey, North Africa	17 (2.0)	23 (5.0)	47 (6.9)
Specified	Surinam, Netherlands Antilles	32 (3.8)	68 (14.9)	95 (14.0)
	Eastern Europe	103 (12.1)	21 (4.6)	73 (10.8)
	Sub-Sahara Africa	26 (3.1)	11 (2.4)	15 (2.2)
	Central and South America	86 (10.1)	22 (4.8)	85 (12.5)
	Rest of Europe	418 (49.2)	211 (46.1)	64 (9.4)
	Asia	83 (9.8)	74 (16.2)	69 (10.2)
	N. America, Canada, Oceania	84 (9.9)	28 (6.1)	231 (34.0)
Number	1–2	527 (13.9)	585 (16.2)	940 (24.7)
of	3–5	674 (17.8)	796 (22.1)	1107 (29.1)
Partners	6–10	570 (15.1)	512 (14.2)	740 (19.5)
	11–20	504 (13.3)	392 (10.9)	388 (10.2)
	21–50	437 (11.5)	236 (6.6)	250 (6.6)
	>50	250 (6.6)	49 (1.4)	338 (8.9)
	Missing	825 (21.8)	1032 (28.7)	37 (1.0)
CLP	Yes	1485 (39.2)	753 (20.9)	872 (22.9)
	Missing	851 (22.5)	2363 (65.6)	2321 (61.1)
Substances	Alcohol	3469 (91.6)	2521 (72.1)	–
w/i 6 mo.	Poppers	1640 (43.3)	1348 (38.6)	–
	Viagra	1081 (28.5)	932 (26.7)	–
	Cannabis	905 (23.9)	634 (18.1)	–
	Ecstasy	708 (18.7)	502 (14.4)	–
	Speed	249 (6.6)	157 (4.5)	–
	Methamphetamine	81 (2.1)	57 (1.6)	–
	GHB	493 (13.0)	408 (11.7)	–
	Cocaine	375 (9.9)	257 (7.4)	–
	Missing	0 (0.0)	107 (3.0)	–
STI	Gonorrhoea	194 (5.2)	174 (5.0)	322 (8.5)
	Chlamydia	260 (7.0)	235 (6.7)	362 (9.5)
	Syphilis	122 (3.3)	136 (3.9)	120 (3.2)
	Hepatitis B	435 (11.5)	383 (11.0)	144 (3.8)
	Hepatitis C	136 (3.6)	56 (1.6)	0 (0.0)
	Herpes	28 (0.7)	34 (1.0)	22 (0.6)
	Warts	102 (2.7)	81 (2.3)	90 (2.4)
	Other STI	–	25 (0.7)	20 (0.5)
	(Skin) diseases	–	2 (0.1)	108 (2.8)
	Missing Mean	32 (0.8)	107 (3.0)	2 (0.1)
HIV status	HIV+	589 (15.6)	414 (11.5)	679 (17.9)
	HIV–	2408 (63.6)	2164 (60.1)	2512 (66.1)
	Never tested	774 (20.4)	864 (24.0)	490 (12.9)
	Missing	16 (0.4)	160 (4.4)	119 (3.1)

*CLP* Condom use with last sexual partner, *GHB* gamma-hydroxybutyraat, *mo*. months, *STI* sexually transmitted diseases, *HIV* human immunodeficiency virus

In all three databases, a majority of MSM was from Amsterdam, had a Dutch ethnicity, used alcohol or poppers in the past 6 months, and was HIV negative. Most questions contained few missing values. However, in SMON and SOAP, condom use with last partner was an optional question, which explains why over 60 % of people did not provide an answer to this question. Concerning our outcomes, approximately a quarter of the participants had one or more STI (EMIS 25 %, SMON 22 %, and SOAP 26 %). Of the HIV tested participants, 589 (19.7 %) were HIV positive in EMIS, 414 (16.1 %) in SMON, and 618 (19.4 %) in SOAP. In addition, a significant fraction of the participants never had an HIV-test (EMIS 20.4 %, SMON 24.0 %, and SOAP 12.9 %).

### Being diagnosed with STI

Multivariable analyses showed that having more partners, using drugs, and being tested for HIV (positive or negative) were associated with being diagnosed with STI in EMIS and SMON (Table 3). Not using condoms with last partner did not reach significance in the multivariable model of SMON, but it did in EMIS. In SOAP, not being Dutch and having used a condom with the last partner were associated with being diagnosed with STI. Specifically, analysing Dutch, non-Dutch Western, and non-Western separately, showed similar odds ratios (OR) 1.0 (95 % CI 0.8–1.4) in EMIS for Western and 1.2 (CI 1.0–1.5) for non-Western respondents, for SMON 1.1 (CI 0.8–1.5) and 1.3 (CI 1.0–1.7) respectively. In SOAP, Western 1.5 (CI 1.2–1.9; the only significant OR) showed higher odds of being diagnosed with STI and non-Western 1.0 (CI 0.8–1.4) did not differ from Dutch respondents.

**Table 3:** Logistic regression analyses of characteristics of participants with STI in EMIS, SMON, and SOAP^a^

	EMIS				SMON				SOAP			
	STI+		Univariable	Multivariable	STI+		Univariable	Multivariable	STI+		Univariable	Multivariable
	No.	%	OR (95 % CI)	aOR (95 % CI)	No.	%	OR (95 % CI)	aOR (95 % CI)	No.	%	OR (95 % CI)	aOR (95 % CI)
Age group (years)^b^												
< 35	242	17.9	1.0	1.0	227	15.1	1.0	1.0	409	26.2	1.0	1.0
35°F49	439	26.9	**1.7 (1.4–2.0)**	1.1 (0.8–1.4)	338	25.5	**1.9 (1.6–2.3)**	0.9 (0.6–1.2)	401	25.0	0.9 (0.8–1.1)	1.0 (0.7–1.3)
> 49	250	31.1	**2.1 (1.7–2.5)**	1.2 (0.9–1.7)	223	28.9	**2.3 (1.9–2.8)**	1.2 (0.8–1.8)	172	27.1	1.0 (0.9–1.3)	0.8 (0.6–1.2)
Residence^b^												
Outside Amsterdam	514	21.8	1.0	1.0	510	18.6	1.0	1.0	586	25.9	1.0	1.0
Amsterdam	318	31.2	**1.6 (1.4–1.9)**	1.0 (0.8–1.3)	255	36.1	**2.5 (2.1–3.0)**	**1.6 (1.1–2.2)**	366	25.5	1.0 (0.8–1.1)	0.9 (0.7–1.2)
Ethnicity												
Dutch	685	24.2	1.0	1.0	671	21.4	1.0	1.0	776	24.9	1.0	1.0
Other	246	25.8	1.0 (0.9–1.3)	1.1 (0.8–1.4)	117	24.8	1.2 (1.0–1.5)	1.0 (0.6–1.5)	206	30.0	**1.3 (1.1–1.6)**	**1.4 (1.0–2.1)**
Number of partners^b^												
1–2	80	15.2	1.0	1.0	83	14.2	1.0	1.0	250	26.6	1.0	1.0
3–5	130	19.3	1.3 (1.0–1.8)	1.1 (0.7–1.6)	151	19.0	**1.4 (1.1–1.9)**	0.8 (0.5–1.4)	261	23.6	0.9 (0.7–1.0)	0.7 (0.5–1.0)
6–10	152	26.7	**2.0 (1.5–2.7)**	1.5 (1.0–2.3)	172	33.6	**3.1 (2.3–4.1)**	1.4 (0.8–2.3)	216	29.2	1.1 (0.9–1.4)	0.9 (0.6–1.3)
11–20	154	30.6	**2.5 (1.8–3.3)**	**1.7 (1.1–2.5)**	141	36.0	**3.4 (2.5–4.6)**	1.6 (0.9–2.7)	88	22.7	0.8 (0.6–1.1)	0.8 (0.5–1.2)
21–50	169	38.7	**3.5 (2.6–4.8)**	**2.1 (1.4–3.2)**	113	47.9	**5.6 (3.9–7.8)**	**2.3 (1.3–4.1)**	58	23.2	0.8 (0.6–1.2)	0.6 (0.3–1.1)
> 50	137	54.8	**6.8 (4.8–9.6)**	**3.4 (2.2–5.2)**	27	55.1	**7.4 (4.0–13.6)**	**3.1 (1.2–8.2)**	103	30.5	1.2 (0.9–1.6)	1.0 (0.6–1.8)
Condom use last partner^b^												
Yes	358	24.1	1.0	1.0	200	26.6	1.0	1.0	235	26.9	1.0	1.0
No	455	31.4	**1.4 (1.2–1.7)**	**1.3 (1.1–1.6)**	167	34.4	**1.4 (1.1–1.9)**	1.1 (0.8–1.5)	126	20.8	**0.7 (0.6–0.9)**	**0.7 (0.6–0.9)**
Drugs												
No	207	12.9	1.0	1.0	196	11.0	1.0	1.0				
Yes 1	218	23.3	**2.1 (1.7–2.5)**	**1.7 (1.3–2.3)**	193	23.3	**2.5 (2.0–3.1)**	1.2 (0.8–1.8)				
Yes > 1	506	40.7	**4.7 (3.9–5.6)**	**2.1 (1.6–2.8)**	399	40.5	**5.5 (4.5–6.7)**	**1.6 (1.1–2.4)**				
HIV^b^												
Never test	55	7.1	1.0	1.0	37	4.3	1.0	1.0	123	25.1	1.0	1.0
HIV+	343	58.2	**18.2 (13.2–25.1)**	**9.0 (5.7–14.3)**	255	61.6	**35.8 (24.4–52.6)**	**17.1 (8.4–35.2)**	161	23.7	0.9 (0.7–1.2)	0.8 (0.5–1.3)
HIV−	531	22.1	**3.7 (2.8–4.9)**	**3.0 (2.0–4.6)**	488	22.6	**6.5 (4.6–9.2)**	**3.8 (2.0–7.4)**	663	26.4	1.1 (0.9–1.3)	1.1 (0.7–1.6)

*95 % CI* 95 % confidence interval, *OR* odds ratio *aOR* adjusted odds ratio, *No.* number, *STI+* diagnosed or self-reported one or more sexually transmitted diseases in the last 12 months

Bold printed statistics differ, *p*-values < .05 are considered significant

^a^Variables are a priori included in multivariable analyses

^b^Factors differ significantly between databases, *p*-values < .05 are considered significant

Inspecting interactions between the variables and database showed an impact of database on the variables age, residence, number of partners, condom use last partner, and HIV status (*p*’s < .01). In EMIS and SMON, being older, living in Amsterdam, having more partners, and been tested for HIV, especially testing positive, increased the odds of being diagnosed with STI. SOAP did not reveal these risk factors. Condom use with the last partner decreased the risk of reporting STI in EMIS (and univariable in SMON). Contrastingly, in SOAP this increased the risk of being diagnosed with an STI.

### Being diagnosed with HIV

Multivariable analyses showed that for both EMIS and SMON, being older, living in Amsterdam (only in EMIS), not using condoms with last partner, using drugs, and being diagnosed with STI were significantly associated with being diagnosed with HIV (Table 4). In contrast, in SOAP only being older, living in Amsterdam, and having more partners were significantly associated with being diagnosed with HIV.

**Table 4:** Logistic regression analyses of characteristics of participants with HIV in EMIS, SMON, and SOAP^a^

	EMIS				SMON				SOAP			
	HIV+		Univariable	Multivariable	HIV+		Univariable	Multivariable	HIV+		Univariable	Multivariable
	No.	%	OR (95 % CI)	aOR (95 % CI)	No.	%	OR (95 % CI)	aOR (95 % CI)	No.	%	OR (95 % CI)	aOR (95 % CI)
Age group (years)												
< 35	100	7.4	1.0	1.0	81	5.7	1.0	1.0	147	9.4	1.0	1.0
35–49	323	19.9	**3.1 (2.4–3.9)**	**1.8 (1.3–2.4)**	216	16.4	**3.2 (2.5–4.2)**	**2.0 (1.2–3.1)**	376	23.5	**3.0 (2.4–3.6)**	**2.5 (1.8–3.6)**
> 49	166	20.7	**3.2 (2.5–4.2)**	**2.4 (1.6–3.4)**	117	15.3	**3.0 (2.2–4.0)**	1.4 (0.8–2.5)	156	24.6	**3.1 (2.5–4.0)**	**4.1 (2.7–6.1)**
Residence												
Outside Amsterdam	275	11.0	1.0	1.0	262	9.8	1.0	1.0	273	12.1	1.0	1.0
Amsterdam	243	23.9	**2.4 (2.0–2.9)**	**1.8 (1.4–2.3)**	147	20.9	**2.4 (1.9–3.0)**	1.2 (0.8–1.8)	396	27.6	**2.7 (2.3–3.3)**	**3.1 (2.3–4.2)**
Ethnicity												
Dutch	437	15.5	1.0	1.0	351	11.6	1.0	1.0	565	18.1	1.0	1.0
Other	152	16.0	1.0 (0.9–1.3)	0.8 (0.6–1.1)	63	13.6	1.2 (0.9–1.6)	1.2 (0.7–2.0)	114	16.6	0.9 (0.7–1.1)	1.1 (0.7–1.8)
Number of partners^b^												
1–2	44	8.4	1.0	1.0	33	5.6	1.0	1.0	122	13.0	1.0	1.0
3–5	90	13.4	**1.7 (1.2–2.5)**	1.7 (1.0–2.8)	81	10.2	**1.9 (1.2–2.9)**	1.8 (0.8–3.8)	154	13.9	1.0 (0.8–1.4)	1.1 (0.7–1.7)
6–10	88	15.5	**2.0 (1.4–2.9)**	1.4 (0.8–2.4)	75	14.6	**2.9 (1.9–4.4)**	1.4 (0.7–3.0)	149	20.1	**1.7 (1.3–2.2)**	**1.7 (1.0–2.7)**
11–20	93	18.5	**2.5 (1.7–3.6)**	1.7 (1.0–2.8)	74	18.9	**3.9 (2.5–6.0)**	1.3 (0.6–2.9)	104	26.8	**2.5 (1.8–3.3)**	**2.0 (1.2–3.4)**
21–50	98	22.5	**3.2 (2.2–4.7)**	1.4 (0.9–2.4)	66	28.0	**6.5 (4.1–10.2)**	1.8 (0.8–4.0)	81	32.4	**3.2 (2.3–4.5)**	**2.2 (1.2–3.9)**
> 50	102	41.0	**7.6 (5.1–11.3)**	**2.7 (1.6–4.7)**	19	38.8	**10.6 (5.4–20.8)**	1.9 (0.6–5.7)	57	16.9	1.4 (1.0–1.9)	**2.2 (1.2–4.0)**
Condom use last partner^b^												
Yes	177	12.0	1.0	1.0	87	11.6	1.0	1.0	146	16.7	1.0	1.0
No	346	23.9	**2.3 (1.9–2.8)**	**2.3 (1.8–2.9)**	145	30.1	**3.3 (2.4–4.4)**	**3.0 (2.1–4.4)**	102	16.8	1.0 (0.8–1.3)	1.0 (0.7–1.4)
Drugs												
No	89	5.6	1.0	1.0	58	3.5	1.0	1.0				
Yes 1	106	11.4	**2.2 (1.6–2.9)**	**1.6 (1.1–2.4)**	74	8.9	**2.7 (1.9–3.9)**	**2.3 (1.2–4.6)**				
Yes > 1	394	31.8	**7.9 (6.2–10.1)**	**3.8 (2.6–5.4)**	282	28.6	**11.2 (8.3–15.1)**	**8.1 (4.4–14.9)**				
STI^b^												
No	246	8.7	1.0	1.0	159	5.9	1.0	1.0	518	18.4	1.0	1.0
Yes 1	198	28.9	**4.3 (3.5–5.3)**	**2.6 (2.0–3.5)**	136	24.3	**5.1 (4.0–6.6)**	**3.8 (2.5–5.7)**	141	17.5	0.9 (0.8–1.2)	0.8 (0.5–1.2)
Yes > 1	145	59.2	**15.3 (11.5–20.4)**	**5.8 (4.1–8.3)**	119	52.2	**17.5 (12.9–23.8)**	**8.9 (5.4–14.8)**	20	11.2	**0.6 (0.4–0.9)**	0.6 (0.3–1.3)

*95 % CI* 95 % confidence interval, *OR* odds ratio, *aOR* adjusted odds ratio, *No.* number, *HIV+* diagnosed or self–reported HIV–positive status

Bold printed statistics differ, *p*–values < .05 are considered significant

^a^Variables are a priori included in multivariable analyses

^b^Factors differ significantly between databases, *p*–values < .05 are considered significant

Inspecting the interactions between the variables and databases showed an effect of database on the number of partners, condom use with last partner, and being diagnosed with STI (*p*’s < .01). Having more partners was associated with being diagnosed with HIV in SOAP, but not in EMIS and SMON. Moreover, not using a condom with their last partner and being diagnosed with STI were associated with being diagnosed with HIV in EMIS and SMON. In SOAP, these relations were lacking.

### Never been tested for HIV

Multivariable analyses showed that being younger, living outside of Amsterdam, and having fewer partners were determinants for never been tested for HIV in all databases (Table 5). Additionally, not using drugs and not being diagnosed with STI were associated with never been tested in EMIS and SMON, but not in SOAP.

**Table 5:** Logistic regression analyses of determinants for ‘never tested for HIV’ in EMIS, SMON, and SOAP^a^

	EMIS				SMON				SOAP			
	NT		Univariable	Multivariable	NT		Univariable	Multivariable	NT		Univariable	Multivariable
	No.	%	OR (95 % CI)	aOR (95 % CI)	No.	%	OR (95 % CI)	aOR (95 % CI)	No.	%	OR (95 % CI)	aOR (95 % CI)
Age group (years)												
< 35	381	28.4	1.0	1.0	476	33.7	1.0	1.0	299	19.1	1.0	1.0
35–49	241	14.8	**0.4 (0.4–0.5)**	0.6 (0.5–0.9)	230	17.5	**0.4 (0.3–0.5)**	0.7 (0.5–1.1)	129	8.1	**0.4 (0.3–0.5)**	**0.4 (0.3–0.6)**
> 49	152	19.0	**0.6 (0.5–0.7)**	0.8 (0.6–1.2)	158	20.6	**0.5 (0.4–0.6)**	0.8 (0.5–1.4)	62	9.8	**0.5 (0.3–0.6)**	**0.4 (0.3–0.7)**
Residence												
Outside Amsterdam	586	25.0	1.0	1.0	738	27.9	1.0	1.0	376	16.6	1.0	1.0
Amsterdam	106	10.4	**0.4 (0.3–0.4)**	**0.5 (0.3–0.6)**	76	10.8	**0.3 (0.2–0.4)**	**0.4 (0.2–0.7)**	88	6.1	**0.3 (0.3–0.4)**	**0.3 (0.2–0.5)**
Ethnicity												
Dutch	623	22.1	1.0	1.0	771	25.4	1.0	1.0	407	13.1	1.0	1.0
Other	151	15.9	**0.7 (0.5–0.8)**	1.2 (0.9–1.6)	93	20.0	**0.7 (0.6–0.9)**	0.9 (0.5–1.6)	83	12.1	0.9 (0.7–1.2)	1.0 (0.6–1.6)
Number of partners^b^												
1–2	151	28.7	1.0	1.0	208	35.6	1.0	1.0	188	20.0	1.0	1.0
3–5	135	20.1	**0.6 (0.5–0.8)**	**0.6 (0.4–0.9)**	194	24.4	**0.6 (0.5–0.7)**	0.7 (0.4–1.1)	180	16.3	0.8 (0.6–1.0)	**0.6 (0.4–0.9)**
6–10	84	14.8	**0.4 (0.3–0.6)**	**0.6 (0.4–0.9)**	67	13.1	**0.3 (0.2–0.4)**	**0.2 (0.1–0.5)**	68	9.2	**0.4 (0.3–0.5)**	**0.3 (0.2–0.5)**
11–20	65	12.9	**0.4 (0.3–0.5)**	**0.6 (0.4–0.9)**	48	12.2	**0.3 (0.2–0.4)**	**0.4 (0.2–0.8)**	17	4.4	**0.2 (0.1–0.3)**	**0.3 (0.2–0.7)**
21–50	43	9.9	**0.3 (0.2–0.4)**	**0.5 (0.3–0.8)**	16	6.8	**0.1 (0.1–0.2)**	**0.2 (0.1–0.5)**	10	4.0	**0.2 (0.1–0.3)**	**0.2 (0.1–0.6)**
> 50	18	7.2	**0.2 (0.1–0.3)**	**0.4 (0.2–0.8)**	3	6.1	**0.1 (0.0–0.4)**	**0.8 (0.2–3.8)**	21	6.2	**0.3 (0.2–0.4)**	**0.2 (0.1–0.4)**
Condom use last partner												
Yes	235	15.9	1.0	1.0	128	17.1	1.0	1.0	133	15.3	1.0	1.0
No	218	15.0	0.9 (0.8–1.1)	1.1 (0.8–1.4)	84	17.5	1.0 (0.8–1.4)	1.4 (0.9–2.0)	77	12.7	0.8 (0.6–1.1)	0.7 (0.5–1.0)
Drugs												
No	485	30.3	1.0	1.0	579	34.4	1.0	1.0				
Yes 1	177	19.1	**0.5 (0.4–0.7)**	0.8 (0.6–1.1)	181	21.8	**0.5 (0.4–0.6)**	**0.6 (0.4–0.9)**				
Yes > 1	112	9.0	**0.2 (0.2–0.3)**	**0.5 (0.4–0.7)**	104	10.5	**0.2 (0.2–0.3)**	**0.4 (0.3–0.7)**				
STI^b^												
No	719	25.3	1.0	1.0	827	30.5	1.0	1.0	367	13.0	1.0	1.0
Yes 1	49	7.2	**0.2 (0.2–0.3)**	**0.3 (0.2–0.5)**	36	6.4	**0.2 (0.1–0.2)**	**0.3 (0.1–0.6)**	98	12.2	0.9 (0.7–1.2)	1.0 (0.6–1.4)
Yes > 1	6	2.4	**0.1 (0.0–0.2)**	**0.2 (0.1–0.4)**	1	0.4	**0.0 (0.0–0.1)**	0.0 (0.0–xx)	25	14.0	1.1 (0.7–1.7)	0.9 (0.4–2.1)

*95 % CI* 95 % confidence interval, *OR* odds ratio, *aOR* adjusted odds ratio, *No.* number, *NT* never having tested for HIV

Bold printed statistics differ, *p*–values < .05 are considered significant

^a^Variables are a priori included in multivariable analyses

^b^Factors differ significantly between databases, *p*-values < .05 are considered significant

Inspecting the interactions between the variables and databases showed an effect of database on the number of partners, and being diagnosed with STI (*p*’s < .03). In SOAP, the relation between having fewer partners and never been tested for HIV seems stronger than in EMIS and SMON. In EMIS and SMON, being diagnosed with STI decreases the chance of never having had an HIV-test, this association was not found in SOAP.

## Discussion

Determinants of being diagnosed with STI or HIV differed remarkably between MSM recruited from STI clinics and MSM participating in internet surveys, whereas the data obtained from the two internet surveys were largely comparable. Moreover, the risk factors found via internet surveys for being diagnosed with STI/HIV are largely similar to findings from previous research [18, 19]. The outcome of having never tested for HIV differed from the outcomes of being diagnosed with STI/HIV. The differences between risk factors between the internet surveys and STI clinic are much less pronounced for this outcome. We will discuss each determinant in the context of the outcomes of this study, the Dutch situation, and previous research.

### Determinants for being diagnosed with STI/HIV and never been tested for HIV

In all three databases, older MSM were more likely to be diagnosed with HIV, as is to be expected. Older MSM will have had more sexual contacts, and are more likely to be exposed to HIV. Moreover, older MSM are also less likely to never been tested for HIV, although multivariably this result only remains for STI clinic data.

Overall, younger and older MSM seem to be underrepresented assuming an equal fraction of MSM over age groups. However, younger MSM (<20 years) might genuinely be a smaller group, because they might not be sexually active or did not ‘come out’ yet. Similarly, there might be less older MSM (>55 years); due to HIV/AIDS or due to the political, legal and cultural climate of their youth [20]. Moreover, the Dutch sample seems somewhat more biased towards older, gay identified, HIV positive MSM as also mentioned in the introduction [10]. Notably, the age distribution is similar in all three databases. Furthermore, in a panel study from 2013, the age of a comparable group of MSM, is even higher than in our databases (mean age 50 years) [21]. This might suggest that people that participating in panel research might be older, but it is also an indication that the higher age of MSM in the Netherlands compared to other countries might not be a bias, but a true representation of the Dutch situation.

MSM from Amsterdam are more likely to be diagnosed with STI/HIV, they are also more likely to be tested for HIV. Because of the large MSM population in Amsterdam, there are more facilities for sexual contacts and testing. More temptations on the one hand, might increase chances to contract STI/HIV. On the other hand, this also affects openness towards and opportunities for testing. A lot of the STI/HIV prevention efforts focus on Amsterdam, and this shows that even though successful (fewer MSM never tested), there might still increased chance to contract STI/HIV. This research suggests that intervention aimed to increase testing uptake could focus more on people not living in Amsterdam, whereas STI and HIV prevention should still be targeted towards MSM in Amsterdam as well.

In the three databases, MSM were either asked about ethnic group, their country of birth, or their cultural background. Surprisingly, the overall composition of ethnicity seems similar over the databases. Indicating that, independent of recruitment method, some ethnic groups were not reached. This included some of the most important minority groups in the Netherlands (i.e., minorities from Turkey, Morocco, Surinam, and the Dutch Antilles). Future research on sexual behaviour should explicitly aim to recruit MSM with specific ethnic backgrounds, or find other ways to investigate behaviour among MSM from the biggest ethnic minority groups in the Netherlands.

Having more partners has divergent influence on being diagnosed with STI/HIV depending on whether data was obtained via internet surveys or from STI clinic attendees, but no difference was found between the databases for having never tested for HIV. Having more partners was a determinant associated with testing, which makes sense as having more partners implies more risk, hence more reasons to test for HIV. Furthermore, having more partners was indicative for being diagnosed with STI but not for being diagnosed with HIV in the internet surveys. STI were reported for the last 12 months, whereas HIV was naturally reported for lifetime, in that light finding an association between the number of partners (over the last 6 or 12 months) for having STI and not for HIV is logical.

Internet surveys and STI clinic data differed for condom use with last sexual partner. Despite the high number of missing data, condom use with the last partner in the internet surveys was protective against STI and HIV, whereas in the STI clinic it was a risk factor for STI and had no effect on HIV positivity. This difference could be explained by timing. Specifically, MSM might visit an STI clinic when there is an indication to do so. Possibly, people visiting an STI clinic suspect a STI, and therefore prospectively used condoms immediately before the visit.

The results for drug use were consistent. Unfortunately, we did not have information on drug use for STI clinic visitors. Using drugs was positively associated with STI diagnosis, diagnoses with HIV, and had tested for HIV. Possibly, drug use influenced risk behaviour directly with drug use resulting in more risky behaviours. Drug use could also influence outcomes indirectly, as people who are more likely to use drugs may also take risk in other domains, because they have an impulsive or sensation-seeking personality [22]. However, risk taking in one domain (doing drugs) is not necessarily indicative for risk taking in other domains [23]. It is important to notice that, frequency of use and the moment of drug use (before or during sexual intercourse) were not assessed. We found similar patterns of behaviour irrespective of the sort of drugs (i.e., uppers, downers, or party drugs); future research should take frequency, sort, and timing of drug use into account. Despite lack of details, drug use still was an important risk factor related to all three outcomes.

Finally, being diagnosed with one or more STI was also associated with being diagnosed with HIV and decreased the chance that MSM had never been tested for HIV. These factors are largely in line with previous studies using EMIS data [18, 19, 24]. In addition, being tested for HIV (and especially being tested positive) increased the risk of being diagnosed with STI.

### Strengths and Limitations

One important limitation of this study is the possible overlap between the databases. The same MSM might have contributed data to all three databases. Moreover, MSM who took part in EMIS after being invited via e-mail the submitted previously to SMON probably filled out both, unfortunately we are unable to identify overlapping records. Since, the internet surveys contained many questions and took some effort to fill out, self-selection bias could have taken place. MSM who are sexually active and attach importance to STI and HIV prevention might be inclined to participate in one or both surveys. Despite our efforts to limit the chances of double participation by our selection of cases in the STI clinic data, we cannot entirely rule out overlap.

Another limitation is that these databases were not designed to be comparable, reflected most notably in difference in question phrasing, content of the questions, and the reference times. Obviously, there have been and still are initiatives for harmonization in the collection of behavioural data within Europe [25]. Notably, even though the surveys are not always comparable, their results are quite similar.

Finally, there is a limited availability of behavioural data in SOAP. This database is intended for surveillance and not research purposes. In the future, we plan to compare additional variables of EMIS and SMON, which were omitted in the current study because they were unavailable in SOAP. It will be possible to look at other psychosocial factors, such as unsafe anal intercourse with steady and casual partners, knowledge, and beliefs [18, 19, 24]. Besides limitations of overlap between the databases, strengths of this research are in the sample sizes of all three databases.

## Conclusion

This research sheds light on the comparability of different recruitment strategies, with the important finding that there seems to be a difference between MSM responding to surveys and MSM visiting STI clinics. Especially when interested in risk factors for contracting STI and HIV, recruitment method seems important. The difference between the internet surveys and STI clinic data is much less pronounced for having never been tested, suggesting both are appropriate for this outcome. The robustness of the risk factors arising from different recruitment methods are limited and show large differences. Finally, this research sheds light on the differences in conclusions drawn based on different data sources, which are pronounced between internet surveys and data from STI clinics.

## Abbreviations

aOR: Adjusted odds ratio
CI: Confidence interval
EMIS: European MSM internet survey
HIV: Human immunodeficiency virus
MSM: Men who have sex with men
OR: Odds ratio
SMON: Schorer Monitor
SOAP: Database STI clinic consultations
STI: Sexually transmitted infection
